# Pre-treatment serum albumin and mutational burden as biomarkers of response to immune checkpoint blockade

**DOI:** 10.1038/s41698-022-00267-7

**Published:** 2022-04-07

**Authors:** Seong-Keun Yoo, Diego Chowell, Cristina Valero, Luc G. T. Morris, Timothy A. Chan

**Affiliations:** 1 Center for Immunotherapy and Precision Immuno-Oncology, Cleveland Clinic, Cleveland, OH 44195 USA; 2 The Precision Immunology Institute, Icahn School of Medicine at Mount Sinai, New York, NY 10029 USA; 3 The Tisch Cancer Institute, Icahn School of Medicine at Mount Sinai, New York, NY 10029 USA; 4 Department of Oncological Sciences, Icahn School of Medicine at Mount Sinai, New York, NY 10029 USA; 5 Department of Surgery, Memorial Sloan Kettering Cancer Center, New York, NY 10065 USA; 6 Cleveland Clinic Lerner College of Medicine, Case Western Reserve University, Cleveland, OH 44106 USA

**Keywords:** Prognostic markers, Cancer, Outcomes research

## Abstract

The effects of cytokine and protein stabilizing carriers, such as serum albumin, on tumor response to immune checkpoint blockade (ICB) is not well understood. By examining 1714 patients across 16 cancer types, we found that high pretreatment serum albumin level predicts favorable tumor radiographic response following ICB treatment in a dose-dependent fashion. Serum albumin is a candidate biomarker that can be combined with tumor mutational burden (TMB) for additional predictive capacity, and the tumor response rate to ICB was ~49% in the albumin-high/TMB-high group.

## Main

The molecular determinants governing systemic immunity states, their stability, and persistence are poorly understood. The balance of cytokines and other immune mediators in the circulation help determine states of inflammation^1,2^. These states have dramatic effects on diseases, such as cancer and autoimmunity. For example, recent studies have shown that soluble molecules, such as interleukin-8^2,3^, interleukin-6^4^, C-reactive protein^4^, and neutrophil-to-lymphocyte ratio (NLR)^5^, can predict ICB outcomes. It is not known how these variables are modulated and how systemic levels influence local tumor killing. However, the combination of these molecules, in addition to tumor genomic factors^6–8^ such as TMB, could work together to determine ICB outcome.

It is well-known that serum albumin stabilizes a broad range of metabolites, hormones, and proteins including cytokines^1,9^. Serum albumin is a prognostic indicator for patients with cancer^10^ and reflects patients’ nutritional status^11^. Furthermore, serum albumin levels may determine systemic inflammation status^1^ and the pharmacokinetics of therapeutic antibodies^12^. Recently, a machine learning approach suggested that pretreatment serum albumin has a great impact on predicting ICB response^13^. We therefore hypothesize that serum albumin may be a key modulator of tumor response from cancer immunotherapies. The effects of serum albumin on clinical response to ICB therapy across diverse cancer types are not fully elucidated. We show that pretreatment serum albumin is a broad and powerful predictor of both radiographic tumor response and patient prognosis following ICB treatment.

To address the effects of pretreatment serum albumin on ICB outcomes, we collected detailed data from 1714 patients treated with ICB across 16 cancer types (Supplementary Table 1). Each patient’s tumor was sequenced via MSK-IMPACT, a next-generation sequencing assay approved by the U.S. Food and Drug Administration (FDA)^14^. For each patient, detailed laboratory and clinical outcomes data were obtained. In patients across 16 cancer types, there was a similar distribution of pretreatment serum albumin concentrations (Supplementary Fig. 1a, b).

**Fig. 1:**
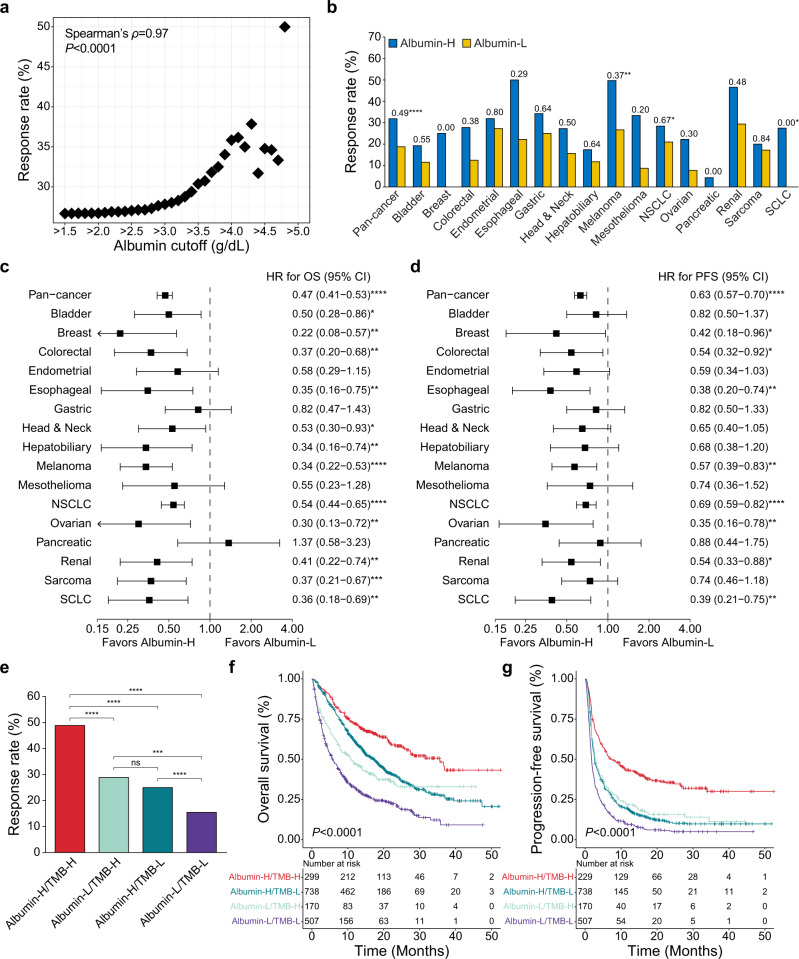
Effects of pretreatment serum albumin on outcomes in patients following immune checkpoint blockade treatment. **a** Radiographic response rate as a function of serum albumin level (pretreatment measurement prior to first infusion). **b** Pan-cancer and subgroup analyses comparing radiographic response rates between the albumin-high (Albumin-H) and -low (Albumin-L) groups. Odds ratios and *χ*^2^ or Fisher’s exact tests *P*-values for each comparison are presented. *P ≤ 0.05 **P ≤ 0.01 ****P ≤ 0.0001. Pan-cancer and subgroup analyses of **c** overall survival (OS) and **d** progression-free survival (PFS). All hazard ratios (HRs) and 95% confidence intervals (CIs) were calculated by univariable analysis. *P*-values were generated by the two-sided log-rank test. *P ≤ 0.05 **P ≤ 0.01 ***P ≤ 0.001 ****P ≤ 0.0001. **e** Combined effects of serum albumin and tumor mutational burden (TMB) on radiographic response rate. *P*-values were generated by the *χ*^2^ test. ns not significant *P ≤ 0.05 **P ≤ 0.01 ***P ≤ 0.001 ****P ≤ 0.0001. Kaplan–Meier plots show the combined effects of serum albumin and TMB on **f** OS and **g** PFS. *P*-values were generated by the two-sided log-rank test.

We determined the best overall response rate as a function of serum albumin level. For this analysis, response rates from patients who have higher pretreatment serum albumin level than each cutoff value were measured. Strikingly, we observed a strong dose-dependent association between radiographic response rate and serum albumin level (Spearman’s *ρ* = 0.97; *P* < 0.0001) (Fig. 1a). We dichotomized the patients into serum albumin-high (Albumin-H) and -low (Albumin-L) groups using the optimal cutoff value (>3.7 g/dL) derived from Youden’s index. We performed four analyses with/without patients with melanoma or non-small cell lung cancer (NSCLC) to confirm if this cutoff value was dependent on them since they are the dominant cancer types in our cohort (Supplementary Fig. 2a–d). All analyses suggested that Youden’s index was maximized at the same optimal cutoff value (>3.7 g/dL). The Albumin-H group showed a clear trend of higher response rates than the Albumin-L group in pan-cancer and subgroup analyses (Fig. 1b). Notably, the Albumin-H group in melanoma, NSCLC, and small cell lung cancer (SCLC) showed significantly higher response rate than the Albumin-L group. This trend was consistent when we used the 50th and the 75th percentile cutoffs (>3.9 g/dL and >4.1 g/dL, respectively) (Supplementary Figs. 3a and 4a).

The Albumin-H group showed superior overall survival (OS) and progression-free survival (PFS) compared to the Albumin-L group (hazard ratio [HR] = 0.47 and 0.63 for OS and PFS, respectively; 95% confidence interval [CI] = 0.41–0.53 and 0.57–0.70 for OS and PFS, respectively; *P* < 0.0001 for both) (Supplementary Fig. 5a, b). These results were still significant when serum albumin was analyzed as a continuous variable and adjusted for several factors associated with ICB treatment outcome^5,7,8,15^ by the multivariable Cox regression analysis (HR = 0.39 and 0.59 for OS and PFS, respectively; 95% CI = 0.34–0.46 and 0.52–0.68 for OS and PFS, respectively; *P* < 0.0001 for both) (Table 1). Importantly, the Albumin-H group showed better outcome independent of performance status (Table 1 and Supplementary Fig. 6a–c). The Albumin-H group had better OS and PFS than the Albumin-L group in most cancer types (Fig. 1c, d). In particular, the Albumin-H group in breast, colorectal, esophageal, melanoma, NSCLC, ovarian, renal, and SCLC showed significantly better OS and PFS than the Albumin-L group. This trend was consistent when we used the 50th percentile cutoff (Supplementary Fig. 3b, c), but some tumor types lost significance when the 75th percentile cutoff was applied (Supplementary Fig. 4b, c).

**Table 1:** Result for multivariable Cox regression analysis.

Variable	Overall survival		Progression-free survival	
	HR (95% Cl)	*P*-value	HR (95% CI)	*P*-value
Serum albumin^a^	0.39 (0.34–0.46)	<0.0001	0.59 (0.52–0.68)	<0.0001
NLR^a^	1.03 (1.02–1.04)	<0.0001	1.02 (1.01–1.02)	<0.0001
TMB^a^	0.98 (0.98–0.99)	<0.0001	0.98 (0.97–0.98)	<0.0001
FCNA^a^	1.84 (1.35–2.50)	<0.0001	1.54 (1.19–2.00)	0.001
Age^a^	1.00 (1.00–1.01)	0.55	0.99 (0.99–1.00)	0.0005
Sex				
Female	Reference		Reference	
Male	0.95 (0.84–1.08)	0.46	0.98 (0.88–1.09)	0.67
BMI				
<30	Reference		Reference	
≥30	0.85 (0.73–0.98)	0.03	0.94 (0.83–1.06)	0.30
Unknown	0.83 (0.12–5.97)	0.85	1.29 (0.32–5.19)	0.72
Stage				
I–III	Reference		Reference	
IV	1.70 (1.21–2.38)	0.002	1.37 (1.08–1.76)	0.01
Unknown	2.05 (1.15–3.66)	0.02	1.45 (0.92–2.28)	0.11
ICB line of treatment				
First line	Reference		Reference	
Subsequent line	1.63 (1.40–1.90)	<0.0001	1.56 (1.38–1.76)	<0.0001
Drug class				
Combo	Reference		Reference	
CTLA-4	1.31 (0.58–2.97)	0.52	0.97 (0.46–2.08)	0.95
PD-1/PD-L1	0.93 (0.77–1.11)	0.41	0.74 (0.74–1.01)	0.07
Cancer type				
Melanoma	Reference		Reference	
NSCLC	1.04 (0.79–1.37)	0.76	0.90 (0.72–1.12)	0.34
Others	1.11 (0.84–1.45)	0.46	0.93 (0.75–1.15)	0.48
Performance status				
ECOG 0	Reference		Reference	
ECOG ≥ 1	1.52 (1.30–1.77)	<0.0001	1.30 (1.15–1.48)	<0.0001
Unknown	1.16 (0.89–1.52)	0.27	0.98 (0.78–1.23)	0.88

*HR* hazard ratio, *CI* confidence interval, *NLR* neutrophil-to-lymphocyte ratio, *TMB* tumor mutational burden, *FCNA* fraction of copy number altered genome, *BMI* body mass index, *ICB* immune checkpoint blockade, *Combo* combination of anti-PD-1/PD-L1 and anti-CTLA-4, *CTLA-4* cytotoxic T-lymphocyte antigen 4, *PD-1* programmed cell death 1, *PD-L1* programmed cell death ligand 1, *NSCLC* non-small cell lung cancer, *ECOG* Eastern Cooperative Oncology Group

^a^Analyzed as continuous values.

We conducted further subgroup analyses using different factors: sex, age, drug class, and TMB (Supplementary Tables 2, 3, 4, and 5). We confirmed the advantage of high serum albumin across the different subgroups, but many of them were not significant due to insufficient sample sizes. However, melanoma and NSCLC still showed significant results in most subgroup analyses.

Moreover, we performed survival analysis using an additional cohort with 5335 patients across 15 cancer types who did not receive ICB treatment (Supplementary Table 6). As expected, we found that high serum albumin (3.7 g/dL) has a positive effect on OS in this cohort. Many cancer types including breast, esophageal, head and neck, melanoma, ovarian, and SCLC showed significantly positive effects of high serum albumin on OS primarily in the cohort with ICB treatment (Supplementary Table 7). Our results show that serum albumin may be informative at multiple levels. Serum albumin is predictive of tumor response to ICB in addition to being generally prognostic for survival.

We examined serum albumin in relation to absolute monocyte count, absolute neutrophil count, NLR, and Systemic inflammation response index (SIRI) to determine if pretreatment serum albumin level is associated with these variables that have been linked to tumor inflammation status. These variables are associated with systemic inflammatory states that can associate with disease states^16–19^ and therapy response^20^. Serum albumin was negatively correlated with all four in pan-cancer and most of the subgroup analyses, which suggests that serum albumin is associated with distinct inflammation states (Supplementary Fig. 7a, b).

Lastly, the combined effects of serum albumin and TMB to predict the patient’s outcomes were evaluated. The TMB-high (TMB-H) and -low (TMB-L) groups were classified based on ≥10 mutations per megabase cutoff, which was approved by the FDA for decision regarding anti-PD-1 therapy^21^. The Albumin-H/TMB-H group had the best OS and PFS as well as radiographic response rate (Fig. 1e–g). The Albumin-H/TMB-H group achieved a 48.83% response rate, which was significantly higher than that of the Albumin-L/TMB-H group (28.82%; OR = 2.35; *P* < 0.0001). Only 15.38% patients of the Albumin-L/TMB-L group showed response to ICB. Median OS and PFS of the Albumin-H/TMB-H group were 35.58 and 7.06 months, respectively, but the Albumin-L/TMB-H group showed only 10.91 and 2.96 months for median OS and PFS, respectively (HR = 0.48 and 0.55 for OS and PFS, respectively; 95% CI = 0.37–0.62 and 0.44–0.68 for OS and PFS, respectively; *P* < 0.0001 for both). Notably, the Albumin-H/TMB-L showed better OS compared with the Albumin-L/TMB-H group (HR = 0.76; 95% CI = 0.61–0.94; *P* = 0.01).

In the present study, we determined the effects of pretreatment albumin on both radiographic tumor response and survival of patients across 16 different cancer types. While it is known that serum albumin may affect overall health and survival^10^, the powerful effect of serum albumin on tumor shrinkage rate from ICB was previously uncharacterized. We also found that pretreatment serum albumin could strongly predict patient outcomes after ICB treatment across most cancer types. It was one of the strongest factors associated with patient survival based on multivariable analysis with other factors known to be related to ICB outcomes. Using a large dataset, we showed that the utility of pretreatment serum albumin is not confined to one or two cancer types but is a broad indicator of ICB outcome. Importantly, serum albumin is a very cost-effective biomarker since it can be acquired from routine blood tests.

Prospective validation will be required to confirm our observations. It is well-known that serum albumin has complex relationships with various biological aspects, such as inflammatory status^1^, nutritional status^11^, and pharmacokinetics^12^. Hence, further studies will need to be done to determine the mechanistic basis of the beneficial effect of serum albumin to ICB treatment.

This study unveils the spectrum of benefits of pretreatment serum albumin in cancer patients with ICB therapy. We propose that pretreatment serum albumin is a cost effective and strong predictor of radiographic response and survival. It can help improve patient stratification when used in combination with TMB. It is also tantalizing to hypothesize that serum albumin infusion may be considered for improving ICB response rates. Our data suggest that serum albumin should be considered as a factor affecting immunotherapy outcomes in future studies.

## Methods

### Patients

This study was approved by the institutional review board of Memorial Sloan Kettering Cancer Center (MSKCC). First, we selected patients with solid tumors diagnosed from 2015 through 2018, who received at least one dose of ICB at MSKCC (*n* = 2827). Then, we excluded patients that did not meet the following criteria: (1) patients with history of only one cancer, (2) patients with laboratory test within 30 days prior to the first dose of ICB, (3) patients not enrolled in blinded trials, (4) patients with cancer types with more than 25 cases in this study. Lastly, we excluded patients who received ICB in the neoadjuvant or adjuvant setting and patients without response data. As a result, 1714 patients across 16 cancer types were analyzed (Supplementary Table 1).

We also evaluated an additional cohort without ICB treatment from a previous study^8^ (*n* = 5335). This cohort was made with the same criteria as the main study cohort, but laboratory tests were conducted within 30 days prior to the first treatment (e.g., surgery, radiotherapy, and chemotherapy). If a patient did not receive any treatment, laboratory test results within 30 days prior to the diagnosis date were used.

### Study outcomes

The study outcomes were OS, PFS, and radiographic response to ICB. OS was calculated from the first infusion of ICB to any cause of death; patients alive at time of review were censored at last contact. For patients who received multiple doses of ICB, the day of first dose was used for the calculation. PFS was analyzed from the first infusion of ICB to disease progression or any cause of death; patients without progression were censored at last attended appointment at MSKCC with any clinician. Best overall response to ICB was measured based on RECIST v1.1 criteria^22^. We also manually reviewed physician notes and imaging studies to classify best overall response for each patient using the same criteria based on change in the sum of diameters of target lesions when formal RECIST evaluation was not available. Responders were defined as patients with complete response or partial response; and non-responders were defined as patients with stable disease or progressive disease.

### Genomic and clinical data

SIRI was calculated as (absolute neutrophil count × absolute monocyte count)/absolute lymphocyte count. We calculated NLR as the absolute neutrophil count divided by the lymphocyte count. Body mass index was calculated as the weight in kilograms divided by the square of the height in meters. All patients provided written informed consent and underwent MSK-IMPACT sequencing, which was approved by the FDA^14^. MSK-IMPACT is a hybridization-capture-based assay, which includes all exons and selected introns of more than 300 cancer related genes (varying across different versions)^14^. TMB was defined as the total number of somatic nonsynonymous mutations per megabase. FACETS software^23^ was used to determine somatic copy number alteration status. We calculated the fraction of copy number altered genome as the sum of the length of | cnlr.median.clust | ≥ 0.2 segments divided by the total length of all segments.

### Statistical analysis

All statistical tests were performed by R programming language (https://www.r-project.org/). Bonferroni corrected pairwise Mann-Whitney U test *P*-values were generated by the “stats” package. The receiver operating characteristic curve, area under the curve, and the optimal cutoff value maximizing Youden’s index were generated by the “pROC” package^24^. Kaplan–Meier plot and Cox proportional hazards model analyses were performed by the “survminer” package.

### Reporting summary

Further information on research design is available in the Nature Research Reporting Summary linked to this article.

## Supplementary information

Supplementary information

REPORTING SUMMARY

## Data Availability

The raw sequencing data are not publicly available because of IRB restrictions. The dataset related to the manuscript will be available from the corresponding author upon reasonable request.

## Code availability

No custom code was used in this study.
