# Supplementary material for: Pre-treatment serum albumin and mutational burden as biomarkers of response to immune checkpoint blockade
Source: NPJ Precis Oncol. 2022 Apr 7;6:23. doi: 10.1038/s41698-022-00267-7 (PMC8990074; doi:10.1038/s41698-022-00267-7)
Supplement: Supplementary file 1 — Supplementary information [file 41698_2022_267_MOESM1_ESM.pdf]

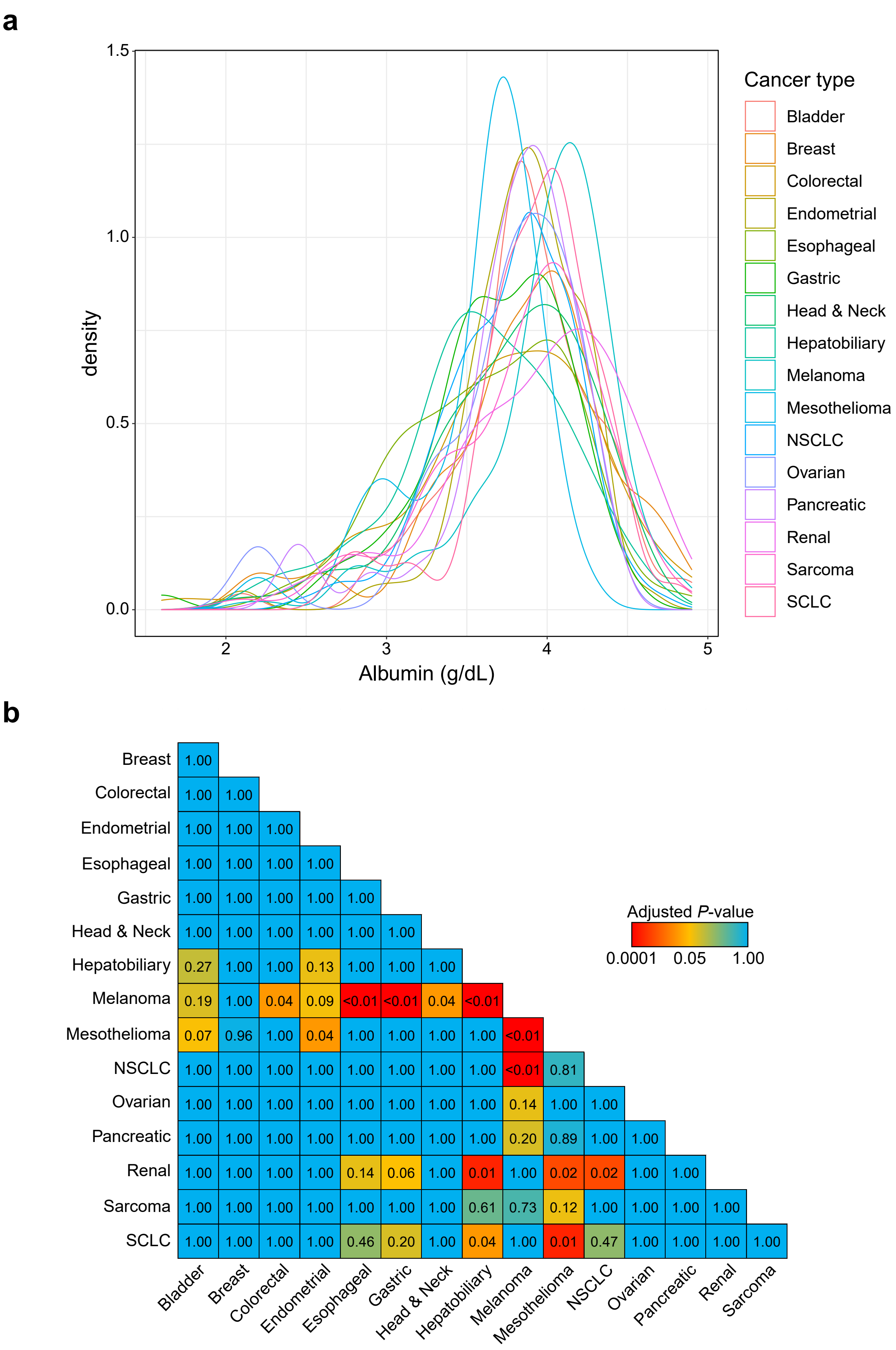

Supplementary Fig. 1: Distribution of pre-treatment serum albumin level across 16 cancer types. **a** Density plots show the distribution of serum albumin levels of each cancer type. **b** Pair-wise Mann-Whitney U test *P*-values among cancer types. *P*-values were corrected by the Bonferroni method. NSCLC and SCLC denote non-small cell lung cancer and small cell lung cancer, respectively.

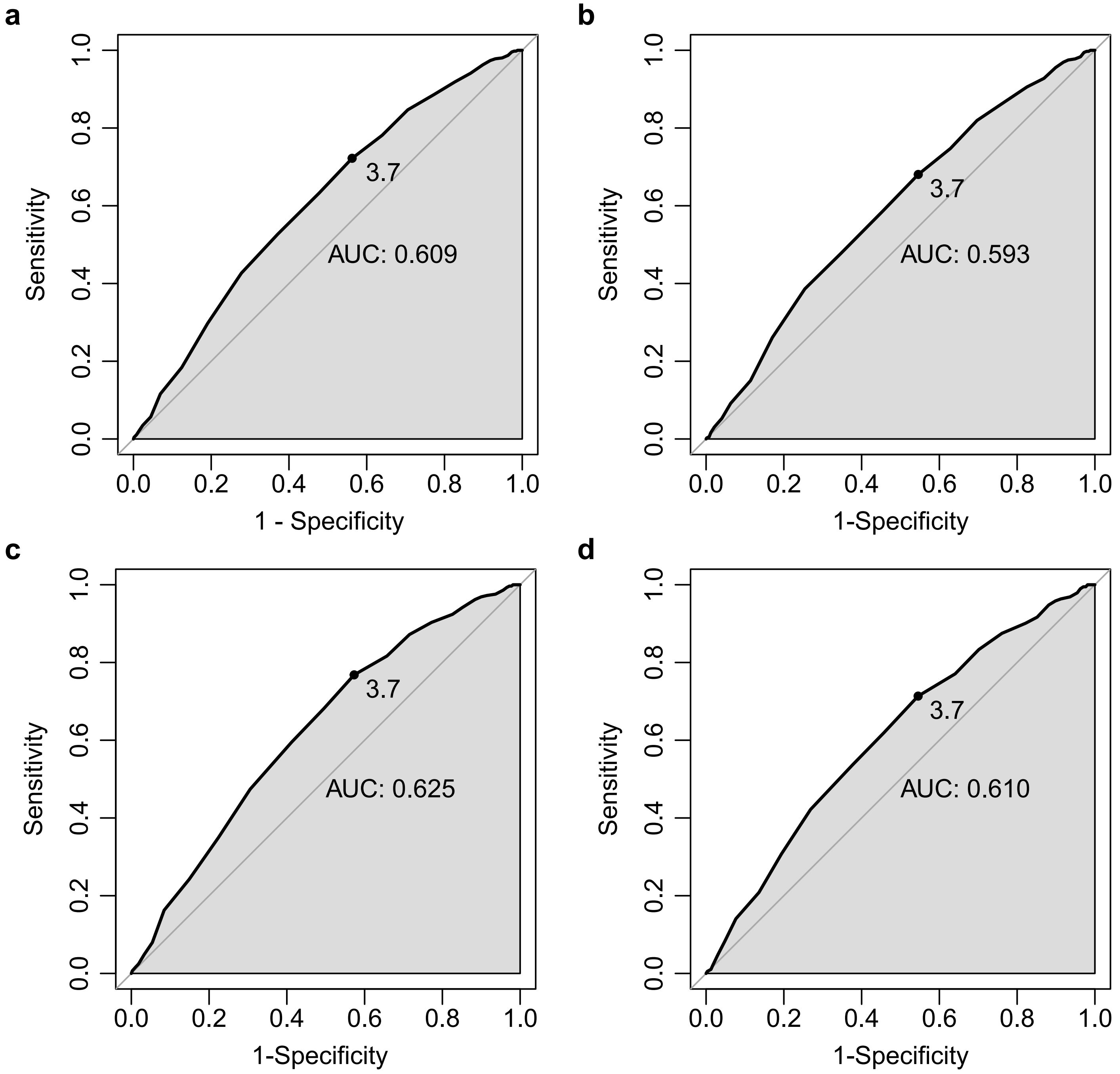

Supplementary Fig. 2: Receiver operating characteristic curve analyses. Youden's index method determined the best cutoff value (>3.7 g/dL) from the curves. Analysis **(a)** with all patients, **(b)** excluding patients with melanoma, **(c)** excluding patients with non-small cell lung cancer (NSCLC), **(d)** excluding patients with melanoma or NSCLC. AUC denotes area under the curve.

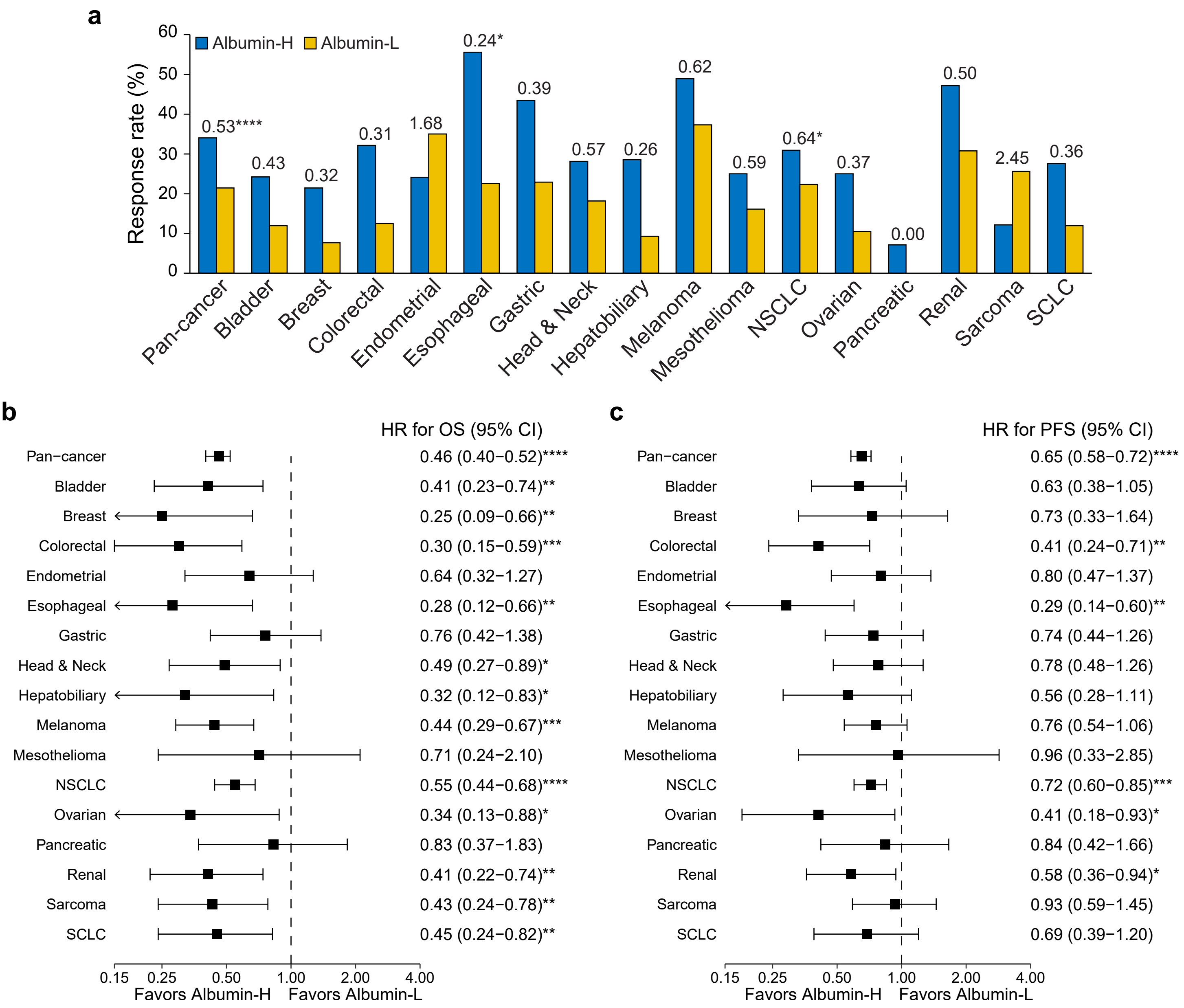

Supplementary Fig. 3: Effect of albumin based on the 50th percentile cutoff (>3.9 g/dL). a Response rate difference between the Albumin-H and Albumin-L groups. Odds ratios and χ^2 or Fisher's exact tests *P*-values for each comparison are presented. Forest plots show **(b)** overall survival (OS) and **(c)** progression-free survival (PFS) difference between two groups. All hazard ratios (HRs) and 95% confidence intervals (CIs) were calculated by univariable analysis. *P*-values were generated by the two-sided log-rank test. * $P \leq 0.05$ ** $P \leq 0.01$ *** $P \leq 0.001$ **** $P \leq 0.0001$.

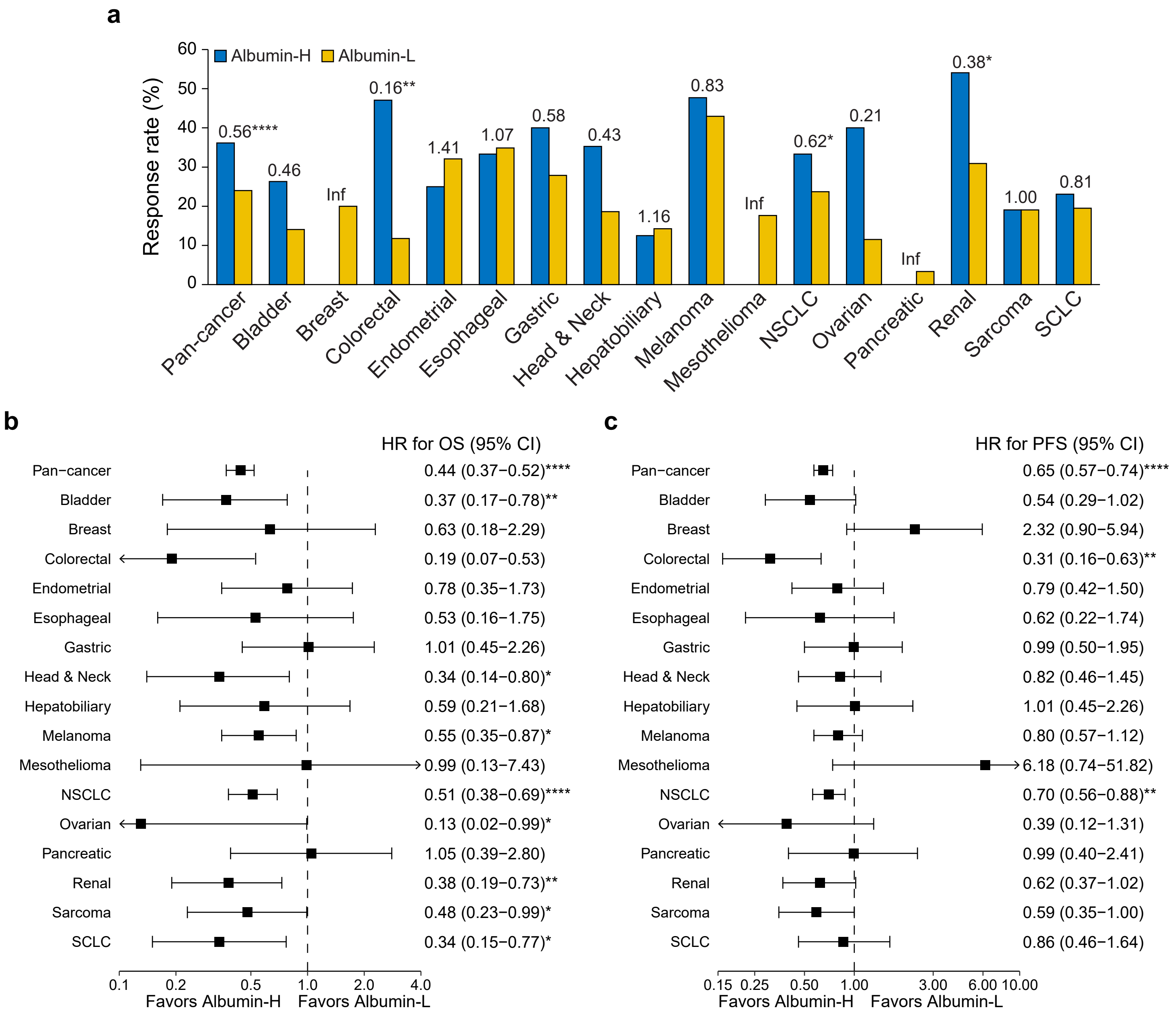

Supplementary Fig. 4: Effect of albumin based on the 75th percentile cutoff (>4.1 g/dL). a Response rate difference between the Albumin-H and Albumin-L groups. Odds ratios and χ^2 or Fisher's exact tests *P*-values for each comparison are presented. Forest plots show **(b)** OS and **(c)** PFS difference between two groups All HRs and 95% CIs were calculated by univariable analysis. *P*-values were generated by the two-sided log-rank test.* *P*≤0.05 ** *P*≤0.01 *** *P*≤0.001 **** *P* ≤0.0001.

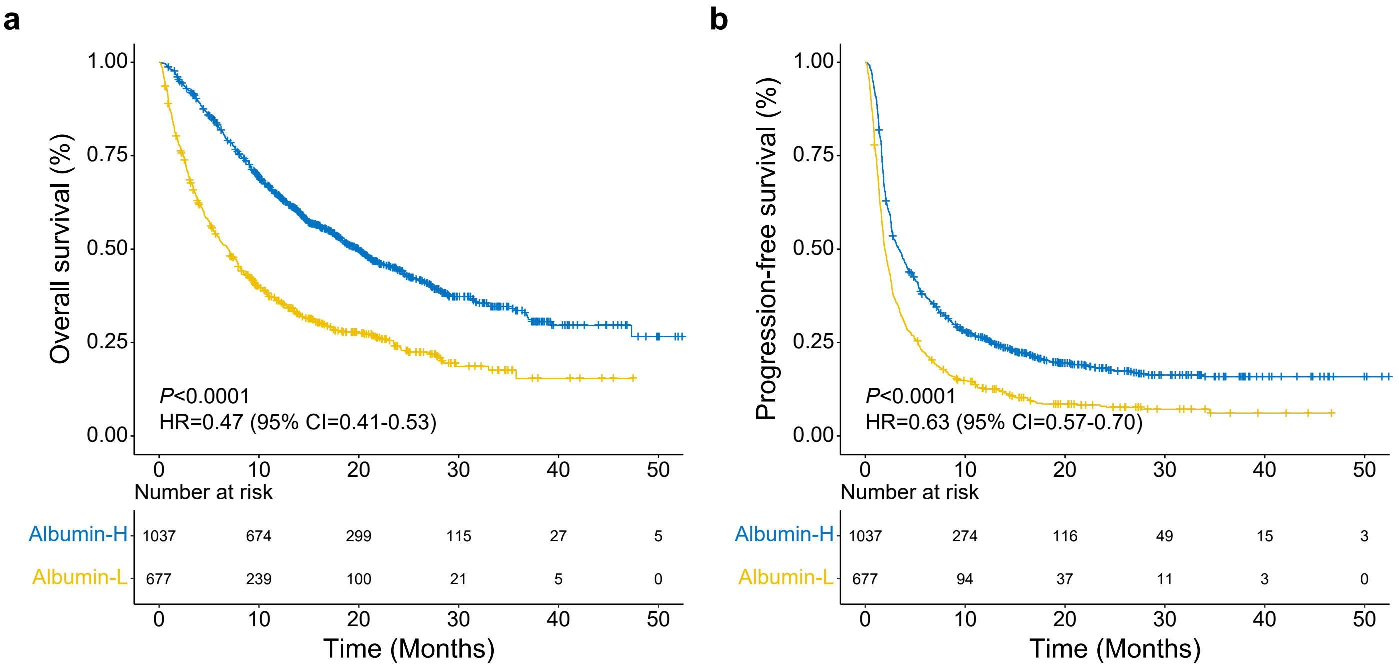

Supplementary Fig. 5: Effect of serum albumin on the patient survival after immune checkpoint blockade therapy. Kaplan-Meier plots show **(a)** OS and **(b)** PFS difference between two groups. The high albumin (Albumin-H) and low albumin (Albumin-L) groups were defined based on the optimal cutoff value (3.7> g/dL). All HRs and 95% CIs were calculated by univariable analysis. *P*-values were generated by the two-sided log-rank test.

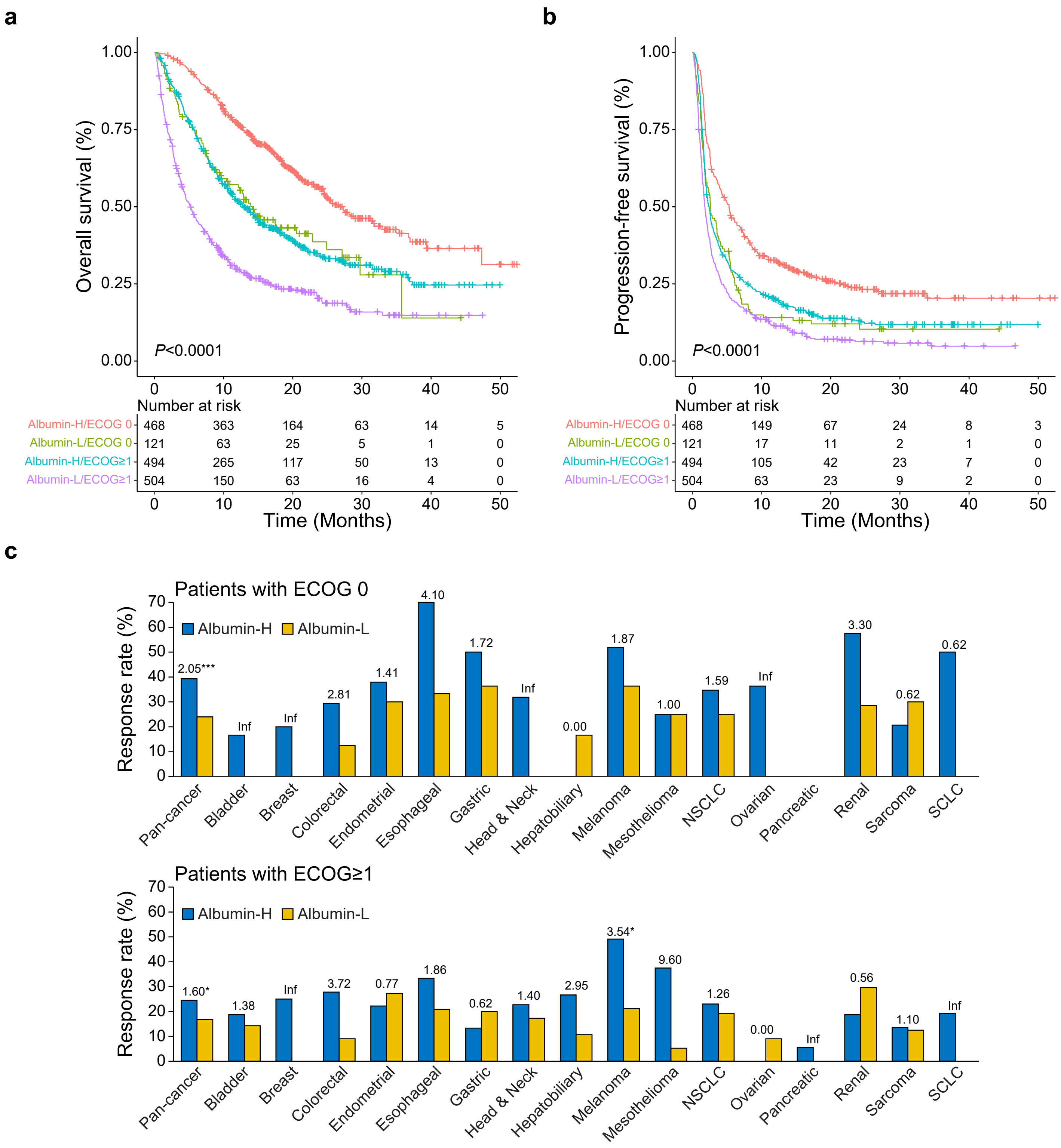

Supplementary Fig. 6: Effect of albumin is independent of Eastern Cooperative Oncology Group (ECOG) performance status. Kaplan-Meier plots showing the combined effects of serum albumin and ECOG performance status on **(a)** OS and **(b)** PFS. *P*-values were generated by the two-sided log-rank test. The Albumin-H and Albumin-L groups were defined based on the optimal cutoff value (3.7> g/dL). c Radiographic response rate between the Albumin-H and Albumin-L groups according to the ECOG performance status. Odds ratios and χ^2 or Fisher's exact tests *P*-values for each comparison are presented. * $P \leq 0.05$ *** $P \leq 0.001$.

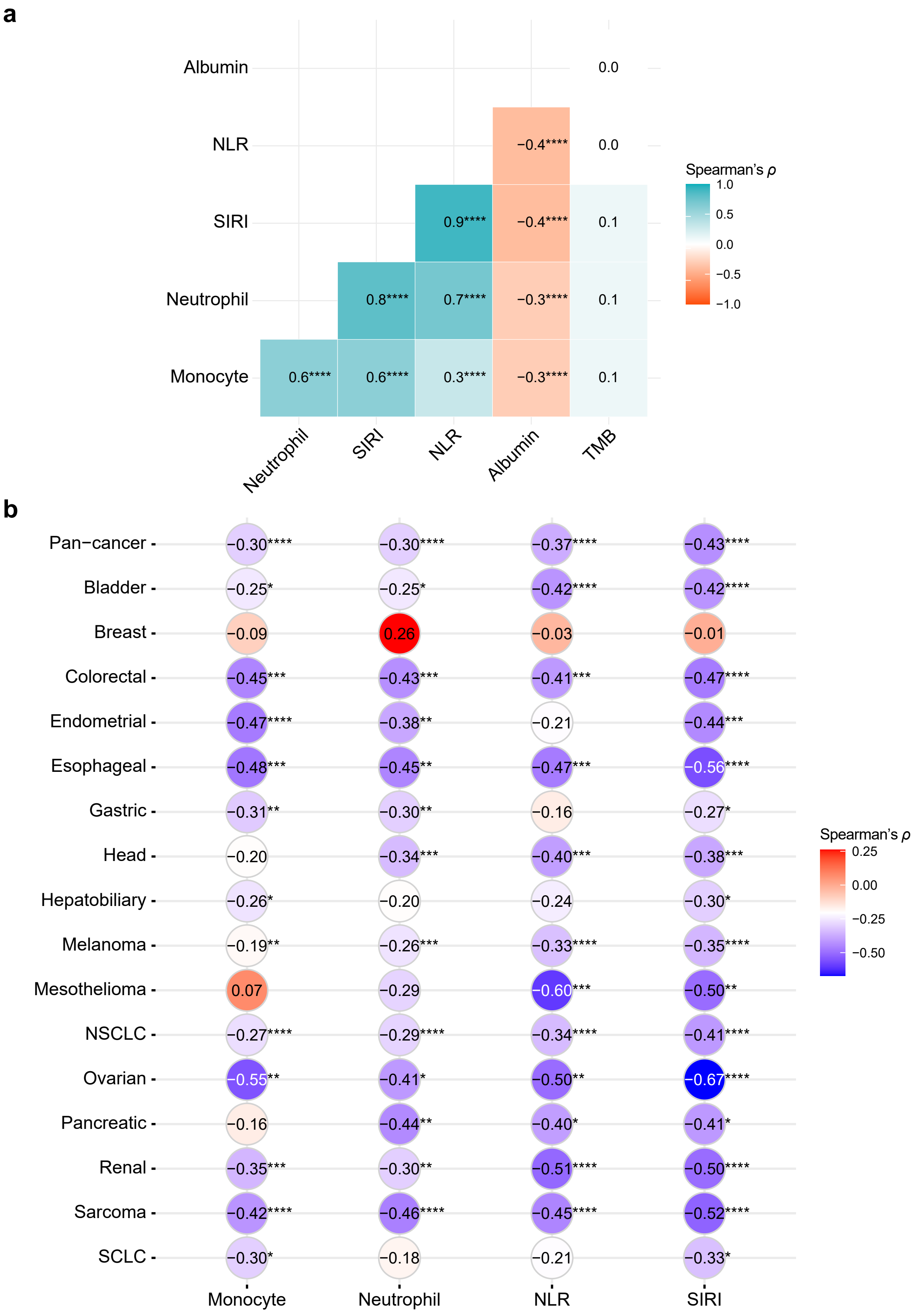

Supplementary Fig. 7: Serum albumin reflects tumor inflammation status. a Correlation matrix plot shows the correlation coefficient among serum albumin, TMB, and four inflammatory markers. NLR and SIRI denote neutrophil-to-lymphocyte ratio and systemic inflammation response index, respectively. **b** Balloon plot shows the correlation coefficient between serum albumin and four inflammatory markers across cancer types. * *P*≤0.05 ** *P*≤0.01 *** *P*≤0.001 **** *P*≤ 0.0001.

Supplementary Table 1 Characteristics of patients with ICB treatment.

Characteristics	No. patients (%)
Sex	
Female	788 (46.0)
Male	926 (54.0)
Age, median, years (IQR)	64 (55-71)
Cancer type	
NSCLC	666 (38.9)
Melanoma	216 (12.6)
Renal	92 (5.4)
Sarcoma	84 (4.9)
Bladder	83 (4.8)
Head & Neck	76 (4.4)
Gastric	71 (4.1)
Endometrial	70 (4.1)
Colorectal	68 (4.0)
Hepatobiliary	57 (3.3)
SCLC	54 (3.2)
Esophageal	49 (2.9)
Pancreatic	36 (2.1)
Mesothelioma	35 (2.0)
Ovarian	30 (1.8)
Breast	27 (1.6)
Drug class	
PD-1/PD-L1	1,422 (83.0)
CTLA-4	7 (0.4)
Combo	285 (16.6)
ICB line of treatment	
First line	522 (30.5)
Subsequent line	1192 (69.5)
Stage	
I-III	105 (6.1)
IV	1,577 (92.0)
Unknown	32 (1.9)
Performance status	
ECOG 0	589 (34.4)
ECOG ≥1	998 (58.2)
Unknown	127 (7.4)

Abbreviations: NSCLC, Non-small cell lung cancer; SCLC, small cell lung cancer; PD-1, programmed cell death 1; PD-L1, programmed cell death ligand 1; CTLA-4, cytotoxic T-lymphocyte antigen 4; Combo, combination of anti-PD-1/PD-L1 and anti-CTLA-4; ICB, immune checkpoint blockade; ECOG, Eastern Cooperative Oncology Group

Supplementary Table 2 Comparison of the effect of high serum albumin (3.7 g/dL) on survival according to the drug class.

Cancer type	Overall survival				Progression-free survival			
	Combo		PD-1/PD-L1 or CTLA-4		Combo		PD-1/PD-L1 or CTLA-4	
	HR (95% CI)	*P*-value	HR (95% CI)	*P*-value	HR (95% CI)	*P*-value	HR (95% CI)	*P*-value
Pan-cancer	0.36 (0.26-0.5)	<0.0001	0.49 (0.43-0.56)	<0.0001	0.53 (0.40-0.69)	<0.0001	1.55 (1.73-1.38)	<0.0001
Bladder	NA	NA	0.58 (0.33-1.02)	0.06	NA	NA	0.99 (0.59-1.67)	0.98
Breast	NA	NA	0.23 (0.09-0.63)	0.004	NA	NA	0.45 (0.19-1.05)	0.07
Colorectal	0.46 (0.08-2.58)	0.38	0.37 (0.19-0.73)	0.004	0.29 (0.07-1.27)	0.10	0.58 (0.33-1.03)	0.06
Endometrial	0.64 (0.09-4.62)	0.66	0.59 (0.28-1.25)	0.17	3.93 (0.42-36.69)	0.23	0.53 (0.29-0.95)	0.03
Esophageal	NA	NA	0.35 (0.16-0.79)	0.01	NA	NA	0.41 (0.21-0.81)	0.01
Gastric	0.31 (0.09-1.04)	0.06	1.09 (0.56-2.12)	0.8	0.49 (0.18-1.38)	0.18	1.03 (0.58-1.84)	0.92
Head & Neck	NA	NA	0.49 (0.28-0.87)	0.01	NA	NA	0.65 (0.40-1.05)	0.08
Hepatobiliary	NA	NA	0.34 (0.16-0.73)	0.006	NA	NA	0.58 (0.32-1.05)	0.07
Melanoma	0.35 (0.17-0.72)	0.004	0.33 (0.19-0.59)	0.0002	0.72 (0.40-1.30)	0.27	0.48 (0.30-0.79)	0.004
Mesothelioma	1.22 (0.20-7.39)	0.83	0.44 (0.16-1.22)	0.11	0.76 (0.12-4.61)	0.76	0.72 (0.32-1.64)	0.44
NSCLC	0.38 (0.21-0.71)	0.002	0.56 (0.45-0.68)	<0.0001	0.59 (0.35-1.01)	0.05	0.70 (0.59-0.83)	<0.0001
Ovarian	NA	NA	0.32 (0.13-0.79)	0.01	NA	NA	0.32 (0.14-0.75)	0.008
Pancreatic	0.29 (0.02-4.65)	0.38	1.45 (0.56-3.75)	0.44	0.29 (0.02-4.65)	0.38	1.01 (0.48-2.12)	0.97
Renal	0.40 (0.04-3.85)	0.43	0.48 (0.26-0.90)	0.02	0.44 (0.05-3.94)	0.46	0.62 (0.37-1.03)	0.06
Sarcoma	0.19 (0.04-0.87)	0.03	0.46 (0.25-0.86)	0.02	0.73 (0.25-2.20)	0.58	0.78 (0.46-1.31)	0.35
SCLC	0.34 (0.14-0.82)	0.02	0.44 (0.16-1.25)	0.13	0.28 (0.12-0.65)	0.003	0.82 (0.30-2.19)	0.69

Abbreviations: Combo, combination of anti-PD-1/PD-L1 and anti-CTLA-4; HR, hazard ratio; CI, confidence interval; NA, not applicable; NSCLC, Non-small cell lung cancer; SCLC, small cell lung cancer

Supplementary Table 3 Comparison of the effect of high serum albumin (3.7 g/dL) on survival according to the age groups.

Cancer type	Overall survival				Progression-free survival			
	Age>64[a]		Age≤64[a]		Age>64 [a]		Age≤64 [a]	
	HR (95% CI)	*P*-value	HR (95% CI)	*P*-value	HR (95% CI)	*P*-value	HR (95% CI)	*P*-value
Pan-cancer	0.51 (0.43-0.61)	<0.0001	0.42 (0.35-0.50)	<0.0001	0.63 (0.54-0.73)	9.48E-10	0.61 (0.52-0.70)	<0.0001
Bladder	0.63 (0.30-1.30)	0.21	0.34 (0.15-0.82)	0.21	0.72 (0.37-1.41)	0.34	0.85 (1.91-0.38)	0.69
Breast	NA	NA	0.28 (0.10-0.77)	0.01	NA	NA	0.49 (1.18-0.20)	0.11
Colorectal	0.27 (0.03-2.26)	0.23	0.34 (0.17-0.67)	0.002	1.06 (0.27-4.15)	0.94	0.37 (0.67-0.21)	0.0009
Endometrial	0.43 (0.19-0.97)	0.04	1.05 (0.28-3.97)	0.95	0.73 (0.37-1.45)	0.37	0.42 (1.10-0.16)	0.08
Esophageal	0.43 (0.10-1.96)	0.28	0.27 (0.10-0.71)	0.008	0.32 (0.09-1.14)	0.08	0.42 (1.02-0.17)	0.05
Gastric	1.05 (0.42-2.63)	0.92	0.69 (0.34-1.41)	0.31	0.74 (0.33-1.65)	0.46	0.80 (1.51-0.42)	0.48
Head & Neck	0.36 (0.13-0.94)	0.04	0.62 (0.30-1.29)	0.20	0.67 (0.31-1.48)	0.32	0.64 (1.18-0.34)	0.15
Hepatobiliary	0.22 (0.05-1.02)	0.05	0.34 (0.13-0.86)	0.02	0.70 (0.28-1.74)	0.44	0.52 (1.18-0.23)	0.12
Melanoma	0.42 (0.23-0.76)	0.004	0.27 (0.14-0.53)	0.0002	0.61 (0.37-1.01)	0.06	0.49 (0.86-0.27)	0.013
Mesothelioma	0.45 (0.16-1.28)	0.14	0.80 (0.18-3.67)	0.78	0.45 (0.17-1.15)	0.09	0.87 (3.96-0.19)	0.86
NSCLC	0.59 (0.46-0.76)	<0.0001	0.47 (0.35-0.64)	<0.0001	0.69 (0.55-0.85)	0.0006	0.69 (0.90-0.53)	0.006
Ovarian	0.39 (0.10-1.59)	0.19	0.16 (0.04-0.56)	0.004	0.55 (0.18-1.69)	0.30	0.16 (0.63-0.04)	0.008
Pancreatic	1.10 (0.28-4.39)	0.89	1.79 (0.58-5.57)	0.32	0.66 (0.20-2.17)	0.49	0.94 (2.27-0.39)	0.90
Renal	0.30 (0.07-1.35)	0.12	0.48 (0.25-0.91)	0.03	0.35 (0.10-1.20)	0.10	0.65 (1.11-0.38)	0.11
Sarcoma	0.36 (0.14-0.91)	0.03	0.38 (0.18-0.79)	0.009	0.67 (0.32-1.44)	0.31	0.75 (1.37-0.41)	0.35
SCLC	0.25 (0.11-0.58)	0.001	0.53 (0.14-1.96)	0.34	0.24 (0.10-0.56)	0.0009	0.91 (2.76-0.30)	0.87

Abbreviations: HR, hazard ratio; CI, confidence interval; NA, not applicable; NSCLC, Non-small cell lung cancer; SCLC, small cell lung cancer; [a]Age groups were divided based on the median age of the cohort

Supplementary Table 4 Comparison of the effect of high serum albumin (3.7 g/dL) on survival according to the sex.

Cancer type	Overall survival				Progression-free survival			
	Male		Female		Male		Female	
	HR (95% CI)	*P*-value	HR (95% CI)	*P*-value	HR (95% CI)	*P*-value	HR (95% CI)	*P*-value
Pan-cancer	0.47 (0.39-0.55)	<0.0001	0.46 (0.39-0.55)	<0.0001	0.65 (0.56-0.75)	<0.0001	0.60 (0.51-0.70)	<0.0001
Bladder	0.54 (0.28-1.04)	0.06	0.42 (0.15-1.20)	0.11	0.94 (0.52-1.71)	0.83	0.62 (0.23-1.63)	0.33
Breast	NA	NA	0.23 (0.09-0.60)	0.003	NA	NA	0.47 (0.21-1.08)	0.08
Colorectal	0.31 (0.10-0.94)	0.04	0.50 (0.23-1.09)	0.08	0.57 (0.24-1.35)	0.20	0.63 (0.31-1.26)	0.19
Endometrial	NA	NA	0.58 (0.29-1.15)	0.12	NA	NA	0.59 (0.34-1.04)	0.07
Esophageal	0.25 (0.10-0.61)	0.002	0.95 (0.19-4.77)	0.95	0.33 (0.15-0.71)	0.005	0.67 (0.18-2.55)	0.56
Gastric	0.55 (0.28-1.10)	0.09	1.45 (0.50-4.19)	0.49	0.79 (0.44-1.43)	0.43	0.88 (0.35-2.20)	0.78
Head & Neck	0.58 (0.30-1.10)	0.1	0.41 (0.1-01.64)	0.21	0.74 (0.43-1.29)	0.29	0.46 (0.15-1.39)	0.17
Hepatobiliary	0.43 (0.16-1.15)	0.09	0.24 (0.07-0.87)	0.03	0.89 (0.41-1.93)	0.78	0.49 (0.20-1.19)	0.12
Melanoma	0.35 (0.20-0.62)	0.0003	0.20 (0.09-0.43)	<0.0001	0.56 (0.36-0.88)	0.01	0.54 (0.26-1.12)	0.10
Mesothelioma	0.45 (0.18-1.14)	0.09	3.46 (0.22-55.78)	0.38	0.54 (0.23-1.26)	0.15	1.42 (0.13-16.04)	0.78
NSCLC	0.53 (0.40-0.70)	<0.0001	0.54 (0.41-0.71)	<0.0001	0.71 (0.56-0.89)	0.004	0.68 (0.54-0.85)	0.001
Ovarian	NA	NA	0.30 (0.13-0.73)	0.007	NA	NA	0.35 (0.16-0.77)	0.01
Pancreatic	1.17 (0.36-3.75)	0.79	1.28 (0.33-4.97)	0.72	0.58 (0.23-1.48)	0.25	1.11 (0.36-3.45)	0.86
Renal	0.38 (0.19-0.79)	0.009	0.42 (0.14-1.26)	0.12	0.63 (0.34-1.16)	0.14	0.39 (0.17-0.92)	0.03
Sarcoma	0.62 (0.27-1.43)	0.26	0.03 (0.01-0.12)	<0.0001	0.96 (0.51-1.80)	0.91	0.36 (0.17-0.75)	0.007
SCLC	0.38 (0.16-0.90)	0.03	0.39 (0.13-1.13)	0.08	0.45 (0.20-1.03)	0.06	0.35 (0.12-1.03)	0.06

Abbreviations: HR, hazard ratio; CI, confidence interval; NA, not applicable; NSCLC, Non-small cell lung cancer; SCLC, small cell lung cancer

Supplementary Table 5 Comparison of the effect of high serum albumin (3.7 g/dL) on survival according to the TMB groups.

Cancer type	Overall survival				Progression-free survival			
	TMB≥10		TMB<10		TMB≥10		TMB<10	
	HR (95% CI)	*P*-value	HR (95% CI)	*P*-value	HR (95% CI)	*P*-value	HR (95% CI)	*P*-value
Pan-cancer	0.48 (0.37-0.62)	<0.0001	0.46 (0.40-0.53)	<0.0001	0.55 (0.44-0.68)	<0.0001	0.67 (0.60-0.76)	<0.0001
Bladder	0.49 (0.16-1.56)	0.23	0.47 (0.25-0.90)	0.02	0.75 (0.28-2.00)	0.56	0.96 (0.53-1.75)	0.9
Breast	NA	NA	0.22 (0.08-0.59)	0.003	NA	NA	0.49 (0.21-1.14)	0.1
Colorectal	0.22 (0.06-0.83)	0.02	0.39 (0.19-0.81)	0.01	0.34 (0.12-0.99)	0.048	0.41 (0.21-0.78)	0.007
Endometrial	0.29 (0.07-1.33)	0.11	0.68 (0.30-1.53)	0.35	0.39 (0.14-1.10)	0.08	0.65 (0.33-1.28)	0.21
Esophageal	NA	NA	0.39 (0.18-0.84)	0.02	NA	NA	0.38 (0.19-0.78)	0.008
Gastric	NA	NA	1.01 (0.56-1.82)	0.98	NA	NA	0.96 (0.57-1.62)	0.89
Head & Neck	0.36 (0.08-1.57)	0.17	0.58 (0.31-1.08)	0.09	0.89 (0.27-2.98)	0.85	0.60 (0.36-1.03)	0.06
Hepatobiliary	2.21 (0.2-24.47)	0.52	0.25 (0.10-0.58)	0.001	3.49 (0.57-21.55)	0.18	0.53 (0.28-0.99)	0.04
Melanoma	0.26 (0.13-0.51)	<0.0001	0.42 (0.23-0.79)	0.007	0.43 (0.25-0.75)	0.003	0.77 (0.46-1.30)	0.33
Mesothelioma	NA	NA	0.57 (0.24-1.34)	0.19	NA	NA	0.74 (0.36-1.55)	0.43
NSCLC	0.70 (0.48-1.01)	0.06	0.48 (0.38-0.60)	<0.0001	0.61 (0.45-0.83)	0.002	0.74 (0.61-0.90)	0.002
Ovarian	NA	NA	0.34 (0.14-0.82)	0.02	NA	NA	0.44 (0.20-0.98)	0.04
Pancreatic	NA	NA	1.47 (0.58-3.72)	0.41	NA	NA	0.79 (0.39-1.63)	0.53
Renal	NA	NA	0.41 (0.22-0.74)	0.003	NA	NA	0.54 (0.33-0.88)	0.01
Sarcoma	NA	NA	0.29 (0.16-0.53)	<0.0001	NA	NA	0.62 (0.38-1.00)	0.048
SCLC	0.52 (0.19-1.45)	0.21	0.25 (0.09-0.66)	0.005	0.49 (0.18-1.32)	0.16	0.24 (0.09-0.62)	0.003

Abbreviations: HR, hazard ratio; CI, confidence interval; NA, not applicable; NSCLC, Non-small cell lung cancer; SCLC, small cell lung cancer

Supplementary Table 6 Characteristics of patients without ICB treatment.

Characteristics	No. patients (%)
Sex	
Female	3,212 (39.8)
Male	2,123 (60.2)
Age at diagnosis, median, years (IQR)	61 (51-70)
Cancer type	
NSCLC	1,087 (20.4)
Colorectal	1,050 (19.7)
Pancreatic	719 (13.5)
Breast	693 (13.0)
Sarcoma	493 (9.2)
Hepatobiliary	281 (5.3)
Ovarian	243 (4.6)
Endometrial	219 (4.1)
Gastric	147 (2.8)
Renal	87 (1.6)
Head & Neck	75 (1.4)
Bladder	72 (1.3)
Esophageal	69 (1.3)
SCLC	59 (1.1)
Melanoma	41 (0.8)
Stage at diagnosis	
I-III	2,752 (51.6)
IV	2,470 (46.3)
Unknown	113 (2.1)

Abbreviations: NSCLC, Non-small cell lung cancer; SCLC, small cell lung cancer

Supplementary Table 7 Comparison of the effect of high serum albumin (3.7 g/dL) on overall survival according to the treatment context.

Cancer type	Without/before ICB treatment		ICB treatment	
	HR (95% CI)	*P*-value	HR (95% CI)	*P*-value
Pan-cancer	0.37 (0.33-0.41)	<0.0001	0.47 (0.41-0.53)	<0.0001
Bladder	0.20 (0.06-0.68)	0.01	0.50 (0.28-0.86)	0.01
Breast	0.58 (0.21-1.59)	0.29	0.22 (0.08-0.57)	0.002
Colorectal	0.34 (0.26-0.45)	<0.0001	0.37 (0.20-0.68)	0.002
Endometrial	0.28 (0.14-0.56)	0.0003	0.58 (0.29-1.15)	0.12
Esophageal	0.69 (0.28-1.67)	0.41	0.35 (0.16-0.75)	0.007
Gastric	0.72 (0.39-1.30)	0.27	0.82 (0.47-1.43)	0.48
Head & Neck	0.43 (0.06-3.29)	0.42	0.53 (0.30-0.93)	0.03
Hepatobiliary	0.55 (0.40-0.75)	0.0002	0.34 (0.16-0.74)	0.006
Melanoma	0.28 (0.03-3.05)	0.29	0.34 (0.22-0.53)	<0.0001
NSCLC	0.24 (0.19-0.30)	<0.0001	0.54 (0.44-0.65)	<0.0001
Ovarian	0.77 (0.43-1.38)	0.37	0.30 (0.13-0.72)	0.007
Pancreatic	0.70 (0.56-0.87)	0.002	1.37 (0.58-3.23)	0.47
Renal	0.08 (0.03-0.19)	<0.0001	0.41 (0.22-0.74)	0.003
Sarcoma	0.39 (0.27-0.55)	<0.0001	0.37 (0.21-0.67)	0.0008
SCLC	0.64 (0.32-1.30)	0.22	0.36 (0.18-0.69)	0.003

Abbreviations: ICB, immune checkpoint blockade; HR, hazard ratio; CI, confidence interval; NSCLC, Non-small cell lung cancer; SCLC, small cell lung cancer
