# Supplementary material for: Pre-treatment serum albumin and mutational burden as biomarkers of response to immune checkpoint blockade
Source: NPJ Precis Oncol. 2022 Apr 7;6:23. doi: 10.1038/s41698-022-00267-7 (PMC8990074; doi:10.1038/s41698-022-00267-7)
Supplement: Supplementary file 2 — REPORTING SUMMARY [file 41698_2022_267_MOESM2_ESM.pdf]

nature portfolio

Corresponding author(s): Timothy A. Chan

Last updated by author(s): Jan 14, 2022

Reporting Summary

Nature Portfolio wishes to improve the reproducibility of the work that we publish. This form provides structure for consistency and transparency in reporting. For further information on Nature Portfolio policies, see our Editorial Policies and the Editorial Policy Checklist.

Statistics

For all statistical analyses, confirm that the following items are present in the figure legend, table legend, main text, or Methods section.

n/a	Confirmed	
☐	☒	The exact sample size (*n*) for each experimental group/condition, given as a discrete number and unit of measurement
☐	☒	A statement on whether measurements were taken from distinct samples or whether the same sample was measured repeatedly
☐	☒	The statistical test(s) used AND whether they are one- or two-sided<br>*Only common tests should be described solely by name; describe more complex techniques in the Methods section.*
☐	☒	A description of all covariates tested
☒	☐	A description of any assumptions or corrections, such as tests of normality and adjustment for multiple comparisons
☐	☒	A full description of the statistical parameters including central tendency (e.g. means) or other basic estimates (e.g. regression coefficient) AND variation (e.g. standard deviation) or associated estimates of uncertainty (e.g. confidence intervals)
☐	☒	For null hypothesis testing, the test statistic (e.g. *F*, *t*, *r*) with confidence intervals, effect sizes, degrees of freedom and *P* value noted<br>*Give P values as exact values whenever suitable.*
☒	☐	For Bayesian analysis, information on the choice of priors and Markov chain Monte Carlo settings
☒	☐	For hierarchical and complex designs, identification of the appropriate level for tests and full reporting of outcomes
☐	☒	Estimates of effect sizes (e.g. Cohen's *d*, Pearson's *r*), indicating how they were calculated

Our web collection on statistics for biologists contains articles on many of the points above.

Software and code

Policy information about availability of computer code

Data collection	No software was used for data collection.
Data analysis	Data analysis was performed using R programming language. No custom code was used in this study.

For manuscripts utilizing custom algorithms or software that are central to the research but not yet described in published literature, software must be made available to editors and reviewers. We strongly encourage code deposition in a community repository (e.g. GitHub). See the Nature Portfolio guidelines for submitting code & software for further information.

Data

Policy information about availability of data

All manuscripts must include a data availability statement. This statement should provide the following information, where applicable:

- Accession codes, unique identifiers, or web links for publicly available datasets
- A description of any restrictions on data availability
- For clinical datasets or third party data, please ensure that the statement adheres to our policy

The raw sequencing data are not publicly available because of IRB restrictions. The dataset related to the manuscript will be available from the corresponding author upon reasonable request.

Field-specific reporting

Please select the one below that is the best fit for your research. If you are not sure, read the appropriate sections before making your selection.

☒ Life sciences ☐ Behavioural & social sciences ☐ Ecological, evolutionary & environmental sciences

For a reference copy of the document with all sections, see nature.com/documents/nr-reporting-summary-flat.pdf

Life sciences study design

All studies must disclose on these points even when the disclosure is negative.

Sample size	1,714 patients across 16 cancer types were analyzed.
Data exclusions	For immunotherapy cohort, we excluded patients did not meet the following criteria: (1) patients with history of only one cancer, (2) patients with complete blood count (CBC) within 30 days prior to the first dose of ICB, (3) patients did not enroll in blinded trials, (4) patients with cancer types with more than 25 cases in this study. Lastly, we excluded patients who received ICB in the neoadjuvant or adjuvant setting and patients without response data.
Replication	Not applicable
Randomization	This study is a retrospective cohort study, no randomization was performed.
Blinding	The process was blinded to patients' serum albumin values. For all experiments the investigators were blinded to group allocation during data collection.

Reporting for specific materials, systems and methods

We require information from authors about some types of materials, experimental systems and methods used in many studies. Here, indicate whether each material, system or method listed is relevant to your study. If you are not sure if a list item applies to your research, read the appropriate section before selecting a response.

Materials & experimental systems

n/a	Involved in the study
☒	☐ Antibodies
☒	☐ Eukaryotic cell lines
☒	☐ Palaeontology and archaeology
☒	☐ Animals and other organisms
☐	☒ Human research participants
☒	☐ Clinical data
☒	☐ Dual use research of concern

Methods

n/a	Involved in the study
☒	☐ ChIP-seq
☒	☐ Flow cytometry
☒	☐ MRI-based neuroimaging

Human research participants

Policy information about studies involving human research participants

Population characteristics	For the cohort with ICB treatment (n=1,714), median age was 64 years (IQR, 55-71); 926 patients (54.0%) were male. For the cohort without ICB treatment (n=5,335), median age was 61 (IQR, 51-70) and 2,123 patients (60.2%) were male.
Recruitment	Based on a dataline request, patients with solid tumors who were first diagnosed during 2015 through 2018, whose tumors underwent next-generation DNA sequencing with MSK-IMPACT, and who received subsequent cancer therapy at Memorial Sloan Kettering Cancer Center were recruited.
Ethics oversight	This study was approved by the institutional review board of Memorial Sloan Kettering Cancer Center.

Note that full information on the approval of the study protocol must also be provided in the manuscript.
